# Link Prediction in Bipartite Nested Networks

**DOI:** 10.3390/e20100777

**Published:** 2018-10-10

**Authors:** Matúš Medo, Manuel Sebastian Mariani, Linyuan Lü

**Affiliations:** 1 Institute of Fundamental and Frontier Sciences, University of Electronic Science and Technology of China, Chengdu 610054, China; 2 Department of Radiation Oncology, Inselspital, Bern University Hospital, University of Bern, 3010 Bern, Switzerland; 3 Department of Physics, University of Fribourg, 1700 Fribourg, Switzerland; 4 URPP Social Networks, Universität Zürich, 8050 Zürich, Switzerland; 5 Alibaba Research Center for Complexity Sciences, Hangzhou Normal University, Hangzhou 311121, China

**Keywords:** link prediction, nested networks, bipartite networks

## Abstract

Real networks typically studied in various research fields—ecology and economic complexity, for example—often exhibit a nested topology, which means that the neighborhoods of high-degree nodes tend to include the neighborhoods of low-degree nodes. Focusing on nested networks, we study the problem of link prediction in complex networks, which aims at identifying likely candidates for missing links. We find that a new method that takes network nestedness into account outperforms well-established link-prediction methods not only when the input networks are sufficiently nested, but also for networks where the nested structure is imperfect. Our study paves the way to search for optimal methods for link prediction in nested networks, which might be beneficial for World Trade and ecological network analysis.

## 1. Introduction

Link prediction is a popular problem in network science [1,2,3,4]. The goal of link prediction is to identify the links that are missing because of erroneous or incomplete data (such as in predicting gene interactions from available data [5]), or links that are likely to appear in the system in the course of its temporal evolution (when we speak of a network that naturally grows or otherwise evolves in time [6,7]). Note that not all networks are equally permissible to link prediction as intrinsic network randomness and link sparsity both contribute to making link prediction more difficult. The attempt to quantify network “link predictability” [8] is highly relevant in this respect.

Various classes of systems have their specificities that impact the link prediction process. For example, social networks typically feature high clustering coefficient (if person A knows B and B knows C, then it is likely that also A knows C), which implies that link prediction methods based on closing such open triangles typically perform well [2,9]. In this sense, the link prediction accuracy depends on the understanding of network specificities, that is, whether the link prediction algorithm can well reflect the corresponding mechanisms of network organization. However, even the most recent reviews of predictions in complex networks [10,11] do not specifically consider link predictions in nested networks. Our goal here is to fill the gap and provide a comparison of how various link prediction methods perform in nested networks.

A network is nested when neighborhoods of low-degree nodes tend to be subsets of neighborhoods of high-degree nodes [12,13]. Nested networks have been much studied with respect to their analysis [14], their formation [15,16,17,18], and their implications [19,20,21]. Nested structures are often found in ecological networks, in particular in plant–animal mutualistic networks [13] and species geographic distribution patterns [12] (see [14,22] for reviews on ecological nested networks), as well as in country-product trade data [23].

Despite the interest of scholars from diverse fields in nestedness, only few attempts [24,25] have been made to exploit the nested topology to identify missing links. Developing effective methodologies for link prediction in nested networks can improve our understanding of at least two important classes of systems which typically exhibit a nested topology: World Trade Networks and ecological mutualistic networks. When analyzing World Trade networks, available data are often characterized by inaccurate or missing information. In the COMTRADE dataset (available at http://comtrade.un.org), for example, given the export of product α from country *i* to *j*, the export volume declared by country *i* often does not match the import volume declared by country *j* for that product [26]. In the recent Economic Complexity research field [23,27], this has led scholars to investigate the robustness of network-based metrics of country fitness and product complexity against network structural perturbation [28,29,30].

Analysis of ecological mutualistic networks is also affected by the fact that unobserved interactions might simply be rare and, therefore, require a longer observation time [31]. Such missing links are fundamentally different from biologically forbidden interactions that cannot physically take place due to species-specific reasons, such as size mismatch or temporal uncoupling [32]. This leads to the problem of estimating sampling bias [33] and its impact on observed network topological properties [34].

Our main contribution is two-fold. First, we provide an extensive benchmarking of link-prediction techniques on synthetic and real data that exhibit a nested structure. In line with recent developments [35,36], we consider not only networks that are overall nested, but also networks that are partitioned into blocks that internally exhibit a nested structure (“in-block nested” networks [36]). Intriguingly, we find that some well-established approaches to link prediction in bipartite networks fail on nested networks. Second, we develop and validate a link-prediction method that takes full advantage of the nested structure of the input data. Importantly, besides achieving optimal performance in perfectly nested networks, the new method performs well also on networks with imperfect nestedness structure, up to a certain number of discrepancies from a perfectly nested network.

## 2. Methods

Before detailing various link prediction methods and their evaluation procedure, we introduce the notation used in this paper. The input bipartite network consists of two sets of nodes with links running only between nodes from different groups. The sizes of the two sets are $N_{1}$ and $N_{2}$, respectively. Nodes in the two groups are labeled with Latin ($i,j,\cdots =1,\cdots ,N_{1}$) and Greek ($\alpha ,\beta ,\cdots =1,\cdots ,N_{2}$) indices, respectively. The network structure can be captured in the $N_{1}\times N_{2}$ biadjacency matrix B with elements $B_{i\alpha }$ ($B_{i\alpha }$ is one if nodes *i* and α are connected, and zero otherwise). The sets of neighbors of nodes *i* and α are $\Gamma_{i}$ and $\Phi_{\alpha }$, respectively. The sizes of these neighborhoods then define the node degree values, $k_{i}:=\left|\Gamma_{i}\right|$ and $d_{\alpha }:=\left|\Phi_{\alpha }\right|$. Finally, the number of links in the network is $E:=\sum_{i}k_{i}=\sum_{\alpha }d_{\alpha }$.

Since the maximal possible number of links in a bipartite network is $N_{1}N_{2}$, there are $N_{1}N_{2}-E$ links that are not present in the input data. In link prediction, we aim to assign score $s_{i\alpha }$, which reflects the link likelihood, to all these links. Links are then ranked by the score in decreasing order; links at the top of this ranking are the most likely candidates for “missing” links.

### 2.1. Link Prediction Methods

#### 2.1.1. Preferential Attachment Index (PrefA)

The number of common neighbors of two nodes is the usual benchmark link prediction method in unipartite networks [2]. However, nodes *i* and α in a bipartite network cannot have any common neighbors by definition, which makes the number of common neighbors as well as popular derived metrics such as the Adamic–Adar index and the Jaccard coefficient [4] not applicable to bipartite networks. The simplest local link prediction metric is thus the preferential attachment index $k_{i}d_{\alpha }$ where the name is of due to the close relation with the preferential attachment mechanism [37]. Notably, this simple approach was found to outperform more sophisticated algebraic link prediction methods based on the eigenvalue decomposition of the network biadjacency matrix B [38].

#### 2.1.2. Number of Local Community Links (LCL)

**Figure 1:**
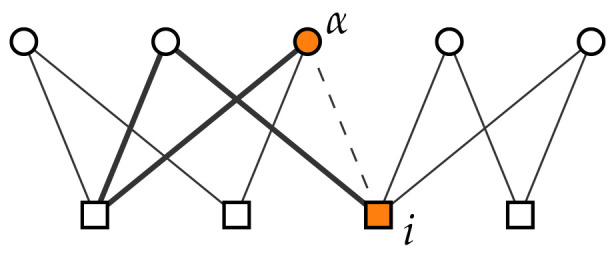
A toy example of a bipartite network where $N_{1}=4$ and $N_{2}=5$. Network links are shown with the solid lines. The dashed line shows the possible link between nodes *i* and α whose likelihood is being evaluated. The thick lines highlight the only existing link between the neighbors of nodes *i* and α (see the prediction method “Number of Local Community Links”). The preferential attachment index (PrefA) and LCL scores of the link between the highlighted nodes are 6 and 1, respectively.

While the common neighbor metric based on paths of length two between the nodes from different sets is not applicable to bipartite networks, paths of length three can exist between nodes from different sets and can be used for link prediction [39]. Since nodes *i* and α have $k_{i}$ and $d_{\alpha }$ neighbors respectively, there can be at most $k_{i}d_{\alpha }$ links that connect them—this quantity is directly used as link prediction score by the preferential attachment (PrefA) index above. The number of local community links, by contrast, takes into account only the actually existing links between the neighbors of nodes *i* and α, and in this way takes the local network structure into account. In Figure 1, for example, nodes *i* and α have comparatively high degree, resulting in high PrefA score of the possible link between them. However, link (i,α) would be a bridge between two otherwise little connected parts of the network. From the point of view of network structure, link (i,α) thus seems little likely which manifests itself in the small number of local community links nodes *i* and α, and a correspondingly low LCL score.

#### 2.1.3. Probabilistic Spreading (ProbS)

Also known under the name *mass diffusion*, probabilistic spreading is a network-based recommendation method [40] that was later generalized in many different ways [41,42]. While the original goal of the method is to produce a *personalized* list of recommended items for each individual user, these personalized recommendation scores can be also used as link prediction scores. In the case of probabilistic spreading, the recommendation score is computed by a step-wise propagation process inspired by random walk on the network. For a given target node *i* (for which the “recommendation” is being computed), unit resource is allocated to all nodes α connected with node *i* (and zero resource otherwise). We denote the initial resource vector as $f_{\alpha }^{\left(i\right)}$ where the superscript highlights the target node *i*. In the first step, the resource propagates along the network’s links to all nodes *j* in the other set of nodes by being divided uniformly among the adjacent nodes. The resulting resource vector $g_{j}^{\left(i\right)}$ can be computed as

(1) $$g_{j}^{\left(i\right)}=\sum_{\alpha ∼j}\frac{f_{\alpha }^{\left(i\right)}}{d_{\alpha }}$$

where the summation is over all α connected with *j* (α∼j). In the second step, the resource propagates along the links again in the same way, yielding the resource vector $h_{\alpha }^{\left(i\right)}$ in the form

(2) $$h_{\alpha }^{\left(i\right)}=\sum_{j∼\alpha }\frac{g_{j}^{\left(i\right)}}{k_{j}}$$

where the sum is now over all *j* connected with α. Score $h_{\alpha }^{\left(i\right)}$ can be then interpreted as the recommendation score of node α when computing recommendation for node *i*. We interpret it here as the link prediction score $s_{i\alpha }$.

#### 2.1.4. Number of Violations of the Nestedness Property (NViol)

In [35], network nestedness was quantified using the concept of violations of the nestedness property. One first defines nodes *i* and *j* as pairwise nested if the following holds: $k_{j}\leq k_{i}:\;\Gamma_{j}\subset \Gamma_{i}$ (note that we adapt here the original notation to bipartite networks). In other words, two nodes are nested if the neighborhood of the higher degree node includes the neighborhood of the other node. Pairwise nestedness can be analogously formulated for nodes from the other set: $d_{\beta }\leq d_{\alpha }:\;\Phi_{\beta }\subset \Phi_{\alpha }$. In [35], they proceed by introducing the number of violations of the nestedness property, which is defined as the number of neighbors of the lower degree node that *are not* neighbors of the higher degree node: $V\left(i,j\right):=\Theta \left(k_{i}-k_{j}\right)\sum_{\alpha }B_{j\alpha }\left(1-B_{i\alpha }\right)$ where the Θ(x) is one for x≥0 and zero otherwise. The total number of violations in the network, *V*, is obtained by summing V(i,j) over all node pairs (i,j).

In social networks where clustering coefficient is high and network links form many triangles, effective link predictions can be obtained [2] by the number of common neighbors which is equal to how many new triangles a new link introduces in the network. Having introduced a measure of network nestedness, we can reason analogously: In a nested network, links that would decrease the number of nestedness violations most are the most likely candidates for missing links. We thus compute the score of link (i,α) as

(3) $$V_{\left(i,\alpha \right)}-V$$

where *V* is the network’s original number of nestedness violations, and $V_{\left(i,\alpha \right)}$ is the number of violations *after* link (i,α) is added. Since *V* is the same for all links in a network, we consider directly $V_{\left(i,\alpha \right)}$ as a link prediction score. Differently from the other link prediction indices, here the lower the better.

Since the value of *V* changes when the biadjacency matrix is transposed, we evaluate NViol on both the original and the transposed network, and choose the approach that yields the best results in terms of AUC (of course, one is free to choose a different criterion).

### 2.2. Evaluation Process

Unlike [43], which considered link prediction in time-stamped country-product data, we do not aim to evaluate the prediction of *future links* in nested networks, because the notion of time is typically not defined in ecological nested networks. Instead, we adopt the usual setting of link prediction in networks without time information, where a small fraction of input data are moved in the probe and the predicted links are then compared with the probe links. In an input network with *E* links, $E_{P}:=f_{P}E$ links are moved in the probe and the remaining $E_{T}:=E-E_{P}$ links comprise training data that are used for prediction; we use $f_{P}=0.1$ here. We remark, though, that the choice of the probe set by timestamps of the network’s links still remains the preferable option in systems where time matters because it exposes link prediction methods to double difficulty of capturing both the systems’ structural patterns, as well as their dynamical patterns [44].

Of the methods included in the evaluation, NViol is the only that is not parameter-free: It has one binary parameter that determines whether it ultimately acts on the original or transposed data. While methods with parameters are best to be evaluated using a triple training-learning-probe division [42] where the additional *learning* set is used to determine the optimal parameter values, we use for simplicity the training-probe division described above because the “minimal” parametrization of NViol is negligible in comparison with the size of the input data. The situation is very different for methods whose number of parameters scales with the input data size (as is the case for the popular matrix factorization recommendation methods [45]) where the simple training-probe evaluation typically substantially overestimates the method’s actual performance.

An obtained ranking of the $N_{1}N_{2}-E_{T}$ links that are not present in the data can be compared with the probe data in various ways [4]. We use four common link performance metrics: Ranking score, AUC, precision, and $F_{1}$ score. To compute the ranking score, *r*, the rank of all probe links in the link prediction list is averaged and subsequently normalized by the list length $N_{1}N_{2}-E_{T}$. Rank score thus lies between zero and one (the lower, the better: small ranking score indicates that the probe items are placed high in the link prediction list). The second metric, AUC, is based on constructing a curve in the unit square [0,1]×[0,1] that corresponds to gradually following the link prediction list from the top (links with the highest score) to the bottom. The coordinates [x,y] of a point on the curve then correspond to the fraction of non-probe and probe-links, respectively, recovered so far. The final AUC value is then obtained by computing the area under thus-constructed curve [46]. AUC lies in the range [0,1]; the higher the value, the better the performance. Note that the ranking score and AUC of a random link prediction list are 0.5. Any result below (in the case of *r*) or above (in the case of AUC) this value thus indicates that link prediction is better than random. The third metric, precision, focuses on the top *L* ranks of the link prediction list. If there are n(L) probe links in the top *L* ranks, we say that the link prediction precision is P(L):=n(L)/L. Precision too lies in the range [0,1] (the higher, the better: precision of one is achieved if all top *L* ranks are occupied by probe links). To evaluate precision, we use L=100. Precision has a closely related counterpart, recall, which is defined as $R\left(L\right):=n\left(L\right)/E_{P}$. The two metrics are combined together in our fourth metric, the $F_{1}$ score, which is defined as the harmonic mean of the observed precision and recall. To make this metric parameter-free, we report the maximum $F_{1}$ score with respect to the number of top ranks *L* included in performance evaluation.

To factor out the randomness of the division into training and probe data, we repeat the evaluation process for 100 random training-probe divisions (in synthetic data, we use 10 independent model realizations and 10 training-probe divisions for each of them), and report the mean and the standard error of the mean for the three chosen evaluation metrics.

## 3. Data

### 3.1. Synthetic Data

**Figure 2:**
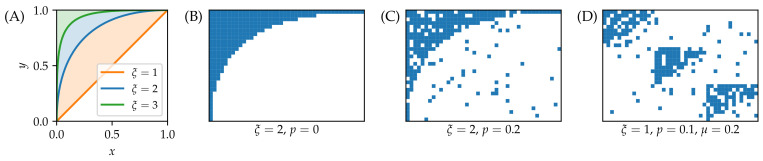
An illustration of synthetic nested networks. (**A**) Nestedness contours for various values of the ξ parameter. (**B**–**D**) Nested networks with $N_{1}=30$ and $N_{2}=42$: Perfectly nested network (**B**), nested network with low noise (**C**), and in-block nested network with three blocks and low noise (parameter values are specified in the panels). The results are averaged over 10 model realizations and 10 independently chosen probe sets for each realization.

To create synthetic nested networks, we adapt to a bipartite setting the construction that was used in [36] to test the detection of simultaneous modular and nested structure in data. We first present this construction in the case without modular structure. The first step is to establish a perfectly nested biadjacency matrix where no nestedness violations are present. To this end, one introduces the contour

(4) $$y=1-{\left(1-x^{1/\xi }\right)}^{\xi }$$

in the unit square [0,1]×[0,1] (see Figure 2); the unit square can be then mapped onto the $N_{1}\times N_{2}$ biadjacency matrix by setting $y:=1-i/N_{1}$ (in this way we follow the usual matrix notation and the first row corresponding to i=1 maps onto the top part of the unit square) and $x:=\alpha /N_{2}$. All biadjacency matrix elements “above” the contour are then set to one, and the remaining elements are to zero. As ξ increases, the degree distribution corresponding to the such-created biadjacency matrix becomes more heterogeneous and the network becomes more sparse. While the resulting biadjacency matrix is nested for any ξ>0, the range ξ∈[1,5] is considered in [36]. After creating a perfectly nested network, noise is introduced by moving each of the initial links with probability *p* to a random node pair (i,α) that is not yet connected by a link. When p=1, the initial nested structure completely disappears and the resulting network is fully random.

The above-described nested networks with noise are in [36] further generalized to *in-block nested* networks where the network consists of $N_{B}$ blocks whose internal connections are more dense than the connections between different blocks (hence the network has a community structure [47]), and each block separately has a nested structure. In the model, this is achieved by forming perfectly nested blocks, adding intrablock noise as described above, and finally adding interblock noise by moving each link with probability $\mu \left(N_{B}-1\right)/N_{B}$ to a randomly chosen node from the original block and a randomly chosen node from another block. When p=μ=0, we obtain unperturbed nested structure in each block and the blocks are mutually disconnected. When μ=1, then the density of links between the blocks is the same as the density of links within each block (i.e., the community structure vanishes). Synthetic data for various model settings are shown in Figure 2.

### 3.2. Real Data

We use six datasets from the ecological networks database http://www.web-of-life.es/ (from the available datasets, all with at least 1000 links are used). These datasets are referred to by their original identifiers (M_SD_022, M_PL_021, M_PL_044, M_PL_015, M_PL_057, and M_PL_062). The five datasets whose identifiers contain PL capture plant-pollinator interactions. M_SD_022 captures fruit-frugivores interactions in two bird communities in south-eastern Brazil.

We further use country-product datasets from two different years (2001 and 2009). These datasets are obtained from the detailed data on the export volumes (measured in dollars) of various categories of products by individual countries (the data has been obtained from https://atlas.media.mit.edu/en/resources/data/). In the first step, the total export of each product category for each country is computed. To represent these data in binary form (a country either exports a product or not), the weighted country-product links are typically filtered [23,26,27] using the concept of *Revealed Comparative Advantage* (RCA) which quantifies how much a country exports a product with respect to the country’s total export and the product’s total export. All country-product pairs with RCA above one are represented as links in the resulting bipartite network (the RCA of one indicates that the country exports as much as one would expect from the country size and the overall export of the given product). The resulting datasets are labeled CP-2001 and CP-2009 (CP stands for country-product, the number denotes the export data year).

Basic statistical properties of the datasets are summarized in Table 1. To quantify the nested structure of the datasets, we use the density of nestedness property violations $\varrho_{V}$ introduced in [35]. This quantity is obtained by diving the actual number of nestedness property violations in the network, *V*, with the maximum number of violations $V_{m}$ where $V_{m}$ is obtained by summing $V_{m}\left(i,j\right):=\Theta \left(k_{i}-k_{j}\right)\min\left(k_{j},N_{2}-k_{i}\right)$ over all node pairs (i,j). To evaluate whether a network has a nested structure, we compare the obtained $\varrho_{V}$ value with the mean $\mu (\varrho_{V})$ and the standard deviation $\sigma (\varrho_{V})$ of $\varrho_{V}$ observed on the network randomized using the classical Configuration Model [37]. The difference $\varrho_{V}-\mu \left(\varrho_{V}\right)$ is negative for all eight real networks (meaning that the original networks are more nested—as measured by $\varrho_{V}$—than their randomized counterparts), and the *z*-score $\left[\varrho_{V}-\mu \left(\varrho_{V}\right)\right]/\sigma \left(\varrho_{V}\right)$ shows the statistical significance (at p<0.05) for six out of eight networks (all except for M_PL_044 and M_PL_062). Note, however, that the observed statistical significance is not indicative of whether these networks are suitable candidates for link prediction with NViol that assumes nested structure in the data. The Country-Product networks, for example, are deemed significantly nested but their values $\varrho_{V}$ and $\mu (\varrho_{V})$ are actually close to each other (they differ by 0.0003).

## 4. Results

**Figure 3:**
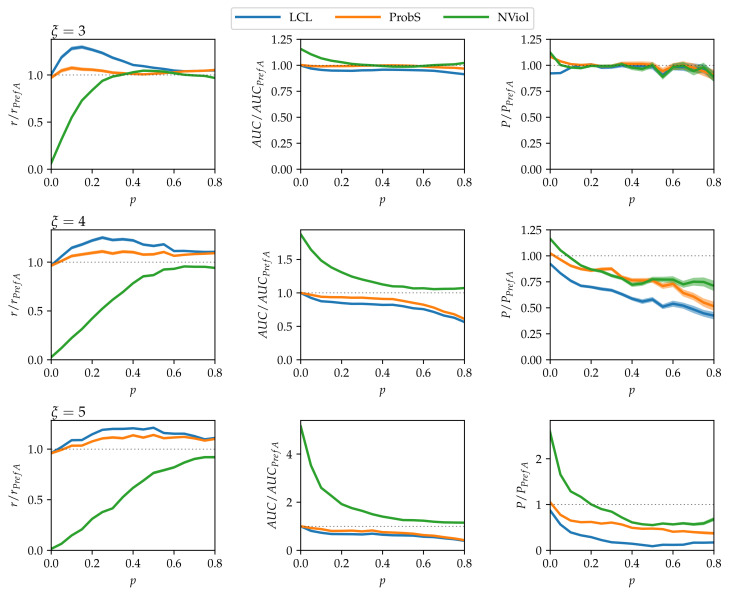
Link prediction results on model data with $N_{1}=100$, $N_{2}=200$, and no community structure. To remove the strong dependency of method performance on the randomization parameter *p*, the shown results are scaled with the results of the simplest PrefA method.

Results on model data with no community structure (B=1) are shown in Figure 3. There is a number of points to note:

1. As ξ grows and the networks’ nested structure thus becomes more pronounced, differences between the methods grow.
2. NViol is generally the best-performing method with respect to the metrics *r* and AUC that take the whole link prediction list into account. Upon a closer inspection of link prediction lists produced by the respective methods, the advantage of NViol is due to its ability to place well also links connecting low-degree nodes that the other methods miss due to their general bias towards high-degree nodes. With NViol, though, probe links adjacent to low-degree nodes are not among the top 100 and hence do not contribute to the method’s precision, yet they rank much better than where other methods are used. If we would increase the number of top ranks included in precision evaluation from 100 to 200 or 300, NViol would have an edge also in this metric.
3. As the randomization parameter *p* grows, PrefA eventually outperforms NViol in terms of link prediction precision. High precision improvement with respect to LCL’s precision for ξ=5 are due to the generally low precision achieved for the sparse networks produced at ξ=5 (which is made further worse by introducing the noise when p>0).
4. ProbS outperforms LCL but lacks behind PrefA and NViol. This is expected because ProbS is based on a “personalized” recommendation algorithm; with no communities in the data, there is no place for personalization and thus ProbS’s merits cannot manifest themselves. The situation becomes radically different when there is more than one nested block in the data (see below).

**Figure 4:**
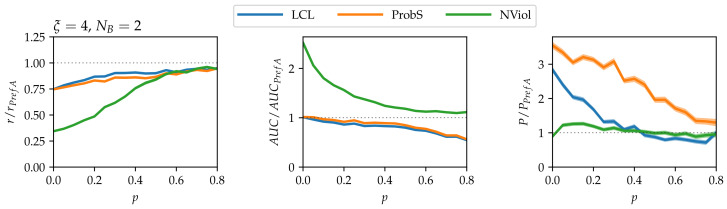
Link prediction results on model data with $N_{1}=100$, $N_{2}=200$, $N_{B}=2$, μ=0 (two blocks, no links between the blocks). As in Figure 3, results are again scaled with the results of the simplest PrefA method.

To illustrate how the relative performance of the methods changes when there is more than one nested block in the data, we choose the simplest case with two blocks and no links between the blocks ($N_{B}=2$, μ=0). As can be seen in Figure 4, the relative performance of PrefA and NViol lowers with respect to the one-block case, in particular in terms of precision which is now worse than the precision achieved by LCL. While NViol remains the best method in terms of the ranking score and AUC, ProbS is clearly best in terms of precision. The reason for the worsening performance of PrefA and NViol is that they both ignore the block structure of the network. If, for example, nodes *i* and α have high degree, PrefA will assign them high score even if they belong to different blocks. By contrast, ProbS and LCL explore the network locally, and thus naturally obey the blocks’ boundaries. To help PrefA and NViol overcome the challenge poised by the presence of multiple blocks, community detection [47] could be first applied to detect blocks in the input network. The very recent method for detecting the structure of nested networks with block-wise nested structure is particularly relevant in this respect [36].

Table 2 summarizes the performance of the evaluated methods on the chosen real datasets. In terms of precision, LCL and ProbS are the best methods. Similarly to Figure 3, global ranking metrics *r* and AUC show a more diverse pattern with NViol being the best method by a large margin in three datasets (ProbS is the best in the remaining ones). The performance gap between the link prediction methods that do not consider the local network structure (PrefA and NViol) and the network neighborhood-based methods (LCL and ProbS) in some networks (M_PL_015 and M_PL_062) suggest that these networks have a more pronounced block structure which puts the PrefA and NViol in disadvantage. Similarly to Figure 4, link prediction with NViol could be helped in these cases by combining it with detection of the block structure.

## 5. Discussion

To summarize, our paper compared the performance of existing link prediction techniques to identify missing links in networks that exhibit nestedness at both macroscopic [14] and mesoscopic [36] scale. For both kinds of structures, we found that a method that exploits the nested structure of such systems (NViol) outperforms existing methods not only for perfectly nested structures, but also for imperfect nested structures up to a certain level of departure from perfect nestedness. The new NViol method performs well also on some real nested datasets where it is the second most successful method after ProbS.

One of the challenges raised by our work is how to best combine the detection of blocks that exhibit an internal nested structure [36] with link prediction. By definition, no links would be predicted between the blocks if the blocks were considered in isolation; however, such rigid assumption is likely to be suboptimal for “mixed” topologies where a given amount of inter-block links exist. Developing a well-performing link-prediction that takes into account this aspect is a challenge for future research.

More generally, our work shows that if we know that a given system exhibit a given structural pattern, we can exploit this information to design a competitive link-prediction techniques. In this respect, we envision that optimal methods for link prediction might be based on a two-step process: First, we learn the topology of the network in hand; second, we adopt a prediction method that is optimal for the detected topology. Once fully developed, such a link prediction method could help us understand how best to measure the degree to which a network is nested. As discussed in Section 3.2, the detection of a statistically significant structural pattern does not necessarily have practical implications. By contrast, whether or not a nestedness-based link prediction method can outperform other benchmark link prediction methods is a very practical way of assessing the value of a network’s nested structure. Work in this direction can help us to better understand not only the nested structure of networks, but also other specific network topologies such as the core-periphery structure, for example.

## Figures and Tables

**Table 1:** Basic properties of the real datasets used to evaluate link prediction methods: Number of rows ($N_{1}$), columns ($N_{2}$), edges (*E*), and the density of edges [$\varrho_{E}:=E/\left(N_{1}N_{2}\right)$].

Dataset	$N_{1}$	$N_{2}$	*E*	$\varrho_{E}$
M_SD_022	207	110	1121	0.05
M_PL_015	131	666	2933	0.03
M_PL_021	91	677	1193	0.02
M_PL_044	110	609	1125	0.02
M_PL_057	114	883	1920	0.02
M_PL_062	456	1044	15,255	0.03
CP-2001	169	781	17,639	0.13
CP-2009	168	774	17,739	0.14

**Table 2:** Mean link prediction results on real datasets. Best performance values for a given method and metric are highlighted with bold. Results are averaged over 100 independently chosen probe sets. Standard error of the mean is less than 0.005 in all cases. If NViol produces best AUC on transposed data, it is labeled as NViol${}^{⊤}$.

M_SD_022						M_PL_057				
method	*r*	AUC	*P*	$F_{1}$		method	*r*	AUC	*P*	$F_{1}$
PrefA	0.20	0.80	0.11	0.13		PrefA	0.38	0.61	0.12	0.10
LCL	0.19	0.80	0.14	0.15		LCL	0.34	0.59	0.14	0.12
ProbS	**0.17**	**0.82**	**0.15**	**0.16**		ProbS	0.33	0.61	**0.16**	**0.13**
NViol ${}^{⊤}$	0.19	0.81	0.11	0.12		NViol	**0.16**	**0.84**	0.12	0.10
M_PL_015						M_PL_062				
method	*r*	AUC	*P*	$F_{1}$		method	*r*	AUC	*P*	$F_{1}$
PrefA	0.23	0.77	0.12	0.09		PrefA	0.20	0.80	0.05	0.05
LCL	0.19	0.80	0.20	0.14		LCL	0.17	0.83	0.12	0.07
ProbS	**0.18**	**0.82**	**0.25**	**0.18**		ProbS	**0.16**	**0.84**	**0.15**	**0.08**
NViol	0.24	0.75	0.13	0.08		NViol ${}^{⊤}$	0.20	0.80	0.04	0.05
M_PL_021						CP-2001				
method	*r*	AUC	*P*	$F_{1}$		method	*r*	AUC	*P*	$F_{1}$
PrefA	0.49	0.49	0.06	0.07		PrefA	0.23	0.78	0.05	0.10
LCL	0.41	0.46	**0.09**	0.09		LCL	0.21	0.79	**0.18**	**0.13**
ProbS	0.40	0.48	**0.09**	**0.10**		ProbS	**0.19**	**0.81**	0.14	0.12
NViol	**0.16**	**0.84**	0.06	0.05		NViol	0.24	0.76	0.06	0.10
M_PL_044						CP-2009				
method	*r*	AUC	*P*	$F_{1}$		method	*r*	AUC	*P*	$F_{1}$
PrefA	0.47	0.52	0.05	0.06		PrefA	0.24	0.77	0.07	0.10
LCL	0.38	0.45	**0.07**	**0.07**		LCL	0.22	0.79	**0.22**	**0.13**
ProbS	0.37	0.46	0.06	**0.07**		ProbS	**0.20**	**0.80**	0.13	0.12
NViol	**0.21**	**0.79**	0.04	0.05		NViol	0.25	0.75	0.08	0.10
